# *FUT1* variants responsible for Bombay or para-Bombay phenotypes in a database

**DOI:** 10.1038/s41598-023-44731-1

**Published:** 2023-10-14

**Authors:** Mikiko Soejima, Yoshiro Koda

**Affiliations:** Department of Forensic Medicine, Kurume University School of Medicine, Kurume, 830-0011 Japan

**Keywords:** Genetics, Clinical genetics, Genotype, Haplotypes

## Abstract

Rare individuals with Bombay and para-Bombay phenotypes lack or have weak expression of the ABO(H) antigens on surface of red blood cells due to no or very weak H-type α(1,2)fucosyltransferase activity encoded by *FUT1.* These phenotypes are clinically important because subjects with these phenotypes can only accept transfusions of autologous blood or blood from subjects with the same phenotypes due to the anti-H antibody. To survey *FUT1* alleles involved in Bombay and para-Bombay phenotypes, the effect of 22 uncharacterized nonsynonymous SNPs in the Erythrogene database on the α(1,2)fucosyltransferase activity were examined by transient expression studies and in silico analysis using four different online software tools. Two nonfunctional alleles (*FUT1* with c.503C>G and c.749G>C) and one weakly functional allele (with c.799T>C) were identified in transient expression studies, while the software predicted that the proteins encoded by more alleles including these would be impaired. Because both nonfunctional *FUT1* alleles appear to link to the nonsecretor alleles, homozygotes of these alleles would be of the Bombay phenotype. The present results suggest that functional assays are useful for characterization of nonsynonymous SNPs of *FUT1* when their phenotypes are not available.

## Introduction

The H blood group antigen is synthesized by α(1,2)fucosyltransferase and is an essential precursor for the synthesis of A and B antigens in the presence of the corresponding A or B transferases^1^. Humans have two types of α(1,2)fucosyltransferase, encoded by *FUT1* and *FUT2*. *FUT1* encodes the H-type α(1,2)fucosyltransferase (H enzyme) which determines expression of the H antigen in the erythroid lineage, whereas *FUT2* encodes a secretor-type α(1,2)fucosyltransferase (Se enzyme) that controls expression of the H antigen in a variety of secretory epithelia and saliva^2,3^. *FUT1* and *FUT2* have approximately 70% DNA sequence similarity and are located 35-kb from each other on chromosome 19q13.3^4–6^.

Two H-deficient red cell phenotypes due to H enzyme deficiency have been recognized: the Bombay phenotype (H– phenotype), in which H, A, and B antigens are completely absent on erythrocytes, saliva, and body fluids, and the para-Bombay phenotype (weak H phenotype; H + w), in which the amount of H antigen (and thereafter, A and B antigens) on erythrocytes is very low. H-deficient red cell phenotypes are extremely rare (the frequency is 1/8000 in Taiwanese with a predominantly para-Bombay phenotype, 1/10,000 in India with a predominantly Bombay phenotype, and 1/1,000,000 in Europe), whereas nonsecretors due to Se enzyme deficiency are present in about 25% of many populations^3,7^. The Bombay phenotype has only nonfunctional alleles of both *FUT1* (*h*) and *FUT2* (*se*), whereas the para-Bombay phenotype has only nonfunctional alleles of *FUT1* (*h*) but at least one functional allele, *FUT2* (*Se*), so that the H antigen produced by the Se enzyme is adsorbed from the serum into the erythrocytes, or has very low H enzyme activity encoded by the weak-functional *FUT1* allele (*H*^*w*^) in the presence of a nonfunctional *FUT2* allele, resulting in negligible H antigen production^1,8,9^. The Bombay phenotype was first recognized by the presence of anti-H in the serum in addition to anti-A and anti-B^10^. Because anti-H produced by subjects with Bombay phenotype carries the risk of severe hemolytic transfusion reactions, subjects with Bombay phenotype require autologous blood donation or blood from other subjects with same phenotype^9,11^. On the other hand, anti-H produced by subjects with para-Bombay phenotype is usually not clinically significant^1^. Therefore, it is clinically important to correctly determine Bombay or para-Bombay phenotypes.

The coding sequence of *FUT1* resides only in exon 4, which encodes a 365-amino acid protein^4,12^. This structural feature of the gene makes it easy to determine the sequences, haplotypes of SNPs of the protein coding region or get expression constructs. Since the cloning of *FUT1*^4^, molecular analysis of H-deficient red cell phenotypes has identified a number of nonfunctional or weak-functional *FUT1* alleles^9,13,14^. At present, the ISBT allele table for the H blood group system (018 H; FUT1FUT2) v6.1 31-MAR-2023 (https://www.isbtweb.org/resource/018h.html) lists 77 *FUT1* alleles, including two functional alleles (FUT1*01 and FUT1*01.02, producing the H + phenotype), 37 alleles involved in phenotype H + weak, i.e., para-Bombay phenotypes (FUT1*01W.01-4, FUT1*01W.05.01 and *0.02*, FUT1*01W.07-24, FUT1*01W.26-29, FUT1*01W.31-39), and 38 alleles involved in phenotype H–, i.e., the Bombay phenotype (FUT1*01N.01-37 and FUT1*0N.01).

However, not every nonsynonymous substitution affects the function of the encoded protein, and we need to estimate the impact of each SNP through in silico analysis in the absence of information on the phenotype^15^, but the prediction results are not always accurate. Alternatively, the enzyme activity has been experimentally determined by transient expression in cultured cells and then measurement of the α(1,2)fucosyltransferase activity by using ^14^C-labeled fucose and its acceptor^13^. Another strategy to examine enzyme activity is flow cytometry of cell surface H antigens by transient expression in cultured cells, which is indirect but does not require a radioisotope^16^. Erythrogene v0.8 (27-Nov-2017) (http://www.erythrogene.com/^17^) extracted the data of blood group alleles from ISBT 018 H (FUT1FUT2) blood group alleles (older version) and the 1000 Genome Project (https://www.internationalgenome.org/^18^) and matched them against blood group reference lists. Seventy-nine alleles are listed for *FUT1*.

In the previous study of *FUT2*, we identified two nonfunctional alleles (*se*) and one weak-secretor allele (*Se*^*w*^) by transient expression studies, but there were discrepancies between the results of transient expression studies and in silico analysis in assessing the functional impacts of each SNP on Se enzyme activity^19^.

In this study, with the aim of determining how many nonsynonymous substitutions affect the activity of the encoding enzyme and whether they could be responsible for the Bombay or para-Bombay phenotypes, we picked 22 *FUT1* alleles from Erythrogene that were not registered in the ISBT database and analyzed their effects on enzyme activity. In addition, three DNA samples with causal substitution (c.725T>G, p.L242R) of the *FUT1* (FUT1*01N.09) giving rise to the classical Indian Bombay phenotype^20,21^ were also examined to better understand the genetic background of this phenotype.

## Materials and methods

### Ethics approval

All methods were carried out in accordance with relevant guidelines and regulations. The oral informed consent was obtained and the DNA samples were taken from participants (47 Bangladeshis in 1999 and 58 Sri Lankan Tamils, 54 Sinhalese in 2002). The statement for oral informed consent approved by ethical committee of Kurume University in 1999 and 2002. However, present study protocol was reviewed and approved by the ethical committee of Kurume University School of Medicine in 2022 using existing and already anonymized DNA (No. 22158, approved date: 31 October 2022).

### DNA samples

Twenty-nine genomic DNAs (HG00118, HG01435, HG01440, HG01443, HG01456, HG01516, HG01577, HG01610, HG01776, HG02003, HG02733, HG02789, HG02870, HG03367, HG03919, HG04185, HG04189 of the 1000 Genomes Project, NA12155 of CEPH/Utah Pedigree 1408, NA18610, NA19019, NA19042, NA19095, NA20289, NA20341, NA20847, NA20887, NA21104, NA21128, NA21141 of International HapMap Project) were purchased from the Coriell Institute for Medical Research (Camden, NJ) (Table 1). Of these, HG02733, HG03919, and HG04189 were registered as having FUT1*01N.09, which is a nonfunctional *FUT1* allele of the classical Indian Bombay phenotype^20,21^. In addition, we used genomic DNA from 58 Sri Lankan Tamils, 54 Sinhalese, and 47 Bangladeshis whose *FUT2* genotypes had already been determined^22,23^.

**Table 1:** Summary of candidates for nonfunctional *FUT1* alleles, their attribution, and evaluation by expression of cell surface H antigens or in silico analyses.

Allele	Expression study (n = 4)			Rs no	Amino acid change	Mutation tester	Mutation assessor (score)	Polyphen2 (score)	SIFT (score)	Coriell no	Attribution
	level (SD)	p vs PC	p vs NC								
c.20G>C	23.9 (7.9)	NS	< 0.001	rs150995632	p.R7P	P	L (1.935)	Probably damaging (0.995)	A (0.01)	NA19019 NA19042	Kenya
c.101A>G	26.5 (5.4)	NS	< 0.001	rs200808269	p.H34R	P	L (1.1)	Benign (0.144)	A (0.04)	HG00118	England
c.181G>A	28.3 (4.9)	NS	< 0.001	rs568231109	p.A61T	P	N (− 0.69)	Benign (0.000)	T (0.96)	HG04185 NA20887	Bangladesh Texas (Gujarati)
c.35C>T c.220C>T	28.7 (3.2)	NS	< 0.001	rs556306430	p.P74S	P	L (0.99)	Benign (0.002)	T (0.41)	NA20847 NA21104	Texas (Gujarati) Texas (Gujarati)
c.229C>G	27.6 (5.3)	NS	< 0.001	rs148719736	p.L77V	P	L (1.225)	Benign (0.039)	T (0.08)	NA20289NA20341	USA (African) USA (African)
c.283C>G	25.5 (5.7)	NS	< 0.001	rs527278015	p.Q95E	P	L (1.21)	Probably damaging (0.99)	T (1.00)	HG02870	Gambia
c.468C>G	27.7 (4.8)	NS	< 0.001	rs543513600	p.D156E	P	N (0.345)	Benign (0.196)	T (0.06)	HG01443	Colombia
c.503C>G	0.4 (0.2)	< 0.001	NS	rs531738794	p.P168R	D	M (3.245)	Probably damaging (1.000)	A (0.00)	HG02789	Pakistan
c.35C>T c.530T>G	24.9 (4.9)	NS	< 0.001	rs564415152	p.L177R	P	M (2.97)	Probably damaging (0.994)	A (0.00)	HG01577	Peru
c.565G>C	27.6 (3.9)	NS	< 0.001	rs182456777	p.D189H	P	M (2.83)	Probably damaging (0.996)	A (0.02)	HG01456	Colombia
c.607C>T	27.4 (5.7)	NS	< 0.001	rs572327966	p.R203C	P	M (3.03)	Benign (0.403)	A (0.00)	HG01610	Spain
c.625G>A	24.7 (5.2)	NS	< 0.001	rs199502509	p.D209N	P	N (0.69)	Benign (0.001)	T (0.18)	NA18610	China
c.35C>T c.649G>A	25.4 (1.6)	NS	< 0.001	rs541722036	p.V217I	D	M (2.37)	Probably damaging (1.000)	A (0.00)	HG02003	Peru
c.691C>T	19.2 (6.7)	0.044	0.002	rs556345040	p.R231C	P	M (2.28)	Probably damaging (1.000)	T (0.09)	HG01435	Colombia
c.725T>G	0.4 (0.3)	< 0.001	NS	rs28934588	p.L242R	D	M (3.215)	Probably damaging (1.000)	A (0.00)	HG04189 HG03919 HG02733	Bangladesh Bangladesh Pakistan
c.749G>C	0.5 (0.2)	< 0.001	NS	rs558351055	p.R250P	D	M (2.91)	Probably damaging (1.000)	A (0.00)	NA21128	Texas (Gujarati)
c.796G>C	24.7 (4.6)	NS	> 0.001	rs200471232	p.E266Q	P	L (1.59)	Possibly damaging (0.571)	T (0.42)	HG01440	Colombia
c.799T>C	2.4 (0.9)	< 0.001	0.011	rs202018483	p.W267R	D	M (2.97)	Probably damaging (0.957)	A (0.00)	NA19095	Nigeria
c.800G>C	10.0 (1.5)	< 0.001	< 0.001	rs548079884	p.W267S	D	M (3.32)	Probably damaging (1.000)	A (0.00)	HG01516	Spain
c.1013T>A	18.8 (5.0)	0.016	< 0.001	rs200387099	p.I338N	D	M (2.745)	Probably damaging (0.986)	A (0.00)	NA12155	Utah
c.1022C>T	19.9 (3.4)	0.009	< 0.001	rs146216905	p.P341L	P	M (3.17)	Probably damaging (1.000)	A (0.00)	HG01776	Spain
c.1064A>G	20.8 (6.7)	NS	< 0.001	rs542146224	p.D355G	D	M (2.725)	Possibly damaging (0.791)	A (0.02)	HG03367	Nigeria
c.1096T>C	27.5 (4.5)	NS	< 0.001	rs562758691	Plus 10 amino acids	–	–	–	–	NA21141	Texas (Indian)

p vs. PC: p value relative to the expression level of positive control (the wild-type allele, FUT1*01, 28.7 ± 3.2%); p vs. NC: p value relative to the expression of the negative control (pcDNA3.1(+) without *FUT1* insert, 0.7 ± 0.2%). *NS* not significant (p > 0.05). P and D represent polymorphism and disease-causing, respectively (MutationTester). N, M, and L represent neutral, medium and low, respectively (MutationAssessor). T and A represent tolerated and affected protein function, respectively (SIFT). In silico analyses were not applicable for 1096T>C as it occurred on T of termination codon TGA.

### Direct sequencing of coding region and haplotype determination of FUT1

The nucleotide sequence is numbered from the A residue of the translation initiation codon as position number 1^4^. The variants were described according to the ISBT guidelines. The coding region of *FUT1* of each genomic DNA was amplified and directly sequenced. For amplification, FUT1-F (5ʹ-GTT CAG AAG CTT CAG TGC ATT TGC TAA TTC GCC TTT C-3ʹ, -39 to -14 bp of *FUT1,* the artificially introduced *Hin*dIII recognition site is underlined) and FUT1-R (5ʹ-CAG GCC TCT GAA GCC ACG TAC T -3ʹ, 1145 to 1166 bp of *FUT1,* the indigenous *Xba*I recognition site is underlined) were used. The 50 µL PCR reaction contained about approximately 7 ng of genomic DNA, 25 µL of PrimeSTAR Max Premix (Takara Bio, Shiga, Japan), and 250 nM of each primer. The PCR temperature conditions were 35 cycles of denaturation at 95 °C for 10 s, annealing at 60 °C for 5 s, and extension at 72 °C for 7 s. Direct Sanger sequencing of the PCR products was performed using each PCR primer as the sequencing primer as described previously^19^. To determine the haplotypes of individuals who were heterozygous at two sites, we cloned PCR products by use of restriction sites of *Hin*dIII and *Xba*I into a mammalian expression vector pcDNA3.1(+) and sequenced the clones. The coding sequence of the *FUT2* of the individuals who were shown to have nonfunctional *FUT1* was also amplified and directly sequenced as described previously^19^.

### Transient expression study to evaluate the effect of each of nonsynonymous SNP of FUT1 on the enzyme activity.

To evaluate the significance of each of nonsynonymous SNP of *FUT1*, transient expression experiments followed by flow cytometry analysis were performed as done with the *FUT2* using an anti-H 1E3 monoclonal antibody^19,24^. In addition to the *FUT1* alleles containing each SNP concerned, the effects of the wild-type allele (FUT1*01), c.725T>G (FUT1*01N.09) inserted into pcDNA3.1(+) vectors were determined. Two μg of each construct together with 60 ng of the pGL3 Promoter was transfected into 2 × 10^5^ COS-7 cells (African green monkey kidney fibroblast-like cell) by means of TransIT-X2 (Mirus Bio LLC, Madison, WI). After 2 days, the cells were immunostained by using a mouse monoclonal antibody to H type 1–4 (1E3)^24^, followed by incubation with FITC-labeled goat anti-mouse IgM (Bethyl Laboratories, Montgomery, TX), and H antigen expression was examined using a BD Accuri C6 system (Becton Dickinson, Franklin Lakes, NJ) as described previously^19^. The experiments were repeated four times independently. The transfection efficiency in each experiment was determined by luciferase luminescence intensity and the similar transfection efficiency was confirmed by the intensity of luciferase light as described previously^19^.

### In silico prediction of effects of nonsynonymous SNPs on H enzyme

The effects of each nonsynonymous SNP of *FUT1* on the function of the enzyme were predicted using four free software programs, MutationTaster (http://www.mutationtaster.org/)^25^, MutationAssessor (http://mutationassessor.org/r3/)^26^, PolyPhen-2 (http://genetics.bwh.harvard.edu/pph2/)^27^, and Sorting intolerant from tolerant (SIFT) (http://sift.jcvi.org/)^28^.

### Screening of c.503C>G, c.725T>G, and c.749G>C in South Asian populations

**Figure 1:**
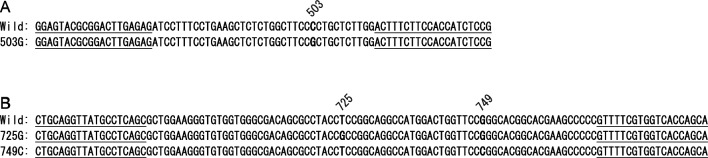
Alignment of DNA sequences of amplified regions for detection of c.503C>G (**A**) and c.725T>G and c.749G>C (**B**) of *FUT1* by HRM analyses. Primer sequences are underlined. DNA sequences of wild type (wild), 503G at 503C>G (503G), 725G at c.725T>G (725G), and 749C at c.749G>C (749C) of *FUT1* are shown.

According to the Erythrogene database, the nonfunctional *FUT1* alleles with c.503C>G c.725T>G, and c.749G>C identified in this study were found in South Asian populations. Therefore, we attempted to screen these substitutions by high resolution melting (HRM)^29^. Primer3 (https://bioinfo.ut.ee/primer3-0.4.0/,^30^) was used to design PCR primers. For detection of c.503C>G, a forward primer (5ʹ-GGA GTA CGC GGA CTT GAG AG-3ʹ, 456–475 bp of *FUT1*) and a reverse primer (5ʹ-CGG AGA TGG TGG AAG AAA GT-3ʹ, 514–533 bp of *FUT1*) were used (Fig. 1A). For detection of both c.725T>G and c.749G>C, a forward primer (5ʹ- CTG CAG GTT ATG CCT CAG C-3ʹ, 673–691 bp of *FUT*), and a reverse primer (5ʹ-TGC TGG TGA CCA CGA AAA C-3ʹ, 769–787 bp of *FUT1*) were used (Fig. 1B).

Real-time PCR and HRM analysis were performed using a LightCycler 480 Instrument II and gene scanning software (Roche Diagnostics, Tokyo, Japan). The 20 μL PCR reaction mixture contained 2–20 ng of genomic DNA, 10 µL of Premix Ex Taq (Probe qPCR) (Takara, Tokyo, Japan), 1 µL of LightCycler 480 High Resolution Melting Dye (Roche Diagnostics, Tokyo, Japan), and 125 nM of each primer. The PCR temperature conditions were 95 °C for 30 s, followed by 40 cycles of 95 °C for 5 s and 60 °C for 20 s. The products were then denatured at 95 °C for 1 min and rapidly cooled to 60 °C for 1 min allowing heteroduplex formation, and data were collected over the range from 74 to 90 °C for c.503C>G or 80–96 °C for c.725T>G and c.749G>C, increasing at 0.02 °C/s with 25 acquisitions/sec. The raw melting curve data were normalized by manual adjustment of linear regions of pre- and post-melt signals of all samples. Temperature shifting was then performed using a temperature shift threshold of default setting (5%) for detection for c.503C>G. On the other hand, temperature shifting was not performed for c.725T>G and c.749G>C to clearly separate heterozygotes of c.725T>G and c.749G>C.

## Results

### Sequence and haplotype determination of FUT1

First, we determined the DNA sequence of the total coding region of the *FUT1* of 29 individuals to survey the nonfunctional alleles of *FUT1* registered in Erythrogene^17^. We confirmed all of the indicated SNPs in respective DNA samples in the database by direct Sanger sequencing of the *FUT1* coding region. As a result, in addition to c.725T>G, we encountered 22 uncharacterized nonsynonymous SNPs: c.20G>C, c.101A>G, c.181G>A, c.220C>T, c.229C>G, c.283C>G, c.468C>G, c.503C>G, c.530T>G, c.565G>C, c.607C>T, c.625G>A, c.649G>A, c.691C>T, c.749G>C, c.796G>C, c.799T>C, c.800G>C, c.1013T>A, c.1022C>T, c.1064A>G, and c.1096T>C.

We then determined haplotypes of four fragments of the *FUT1* coding region with two substitutions, that is, c.35C>T and c.181G>A, c.35C>T and c.220C>T, c.35C>T and c.649G>A, c.822C>A and c.1064A>G, by subcloning them into plasmids. Sequencing of the clones revealed that only one haplotype of the four alleles differed from those listed in the database. That is, c.822C>A and c.1064A>G were on the same chromosome in the database, but each was on a different chromosome. On the other hand, consistent with the database, c.181G>A, c.220C>T, and c.649G>A were on the functional *FUT1* allele with c.35C>T (FUT1*01.02). Because c.822C>A is a synonymous SNP, we performed functional analyses of the 23 alleles including c.725T>G (FUT1*01N.09) listed in Table 1.

### Functional analyses of candidates of nonfunctional FUT1 alleles

**Figure 2:**
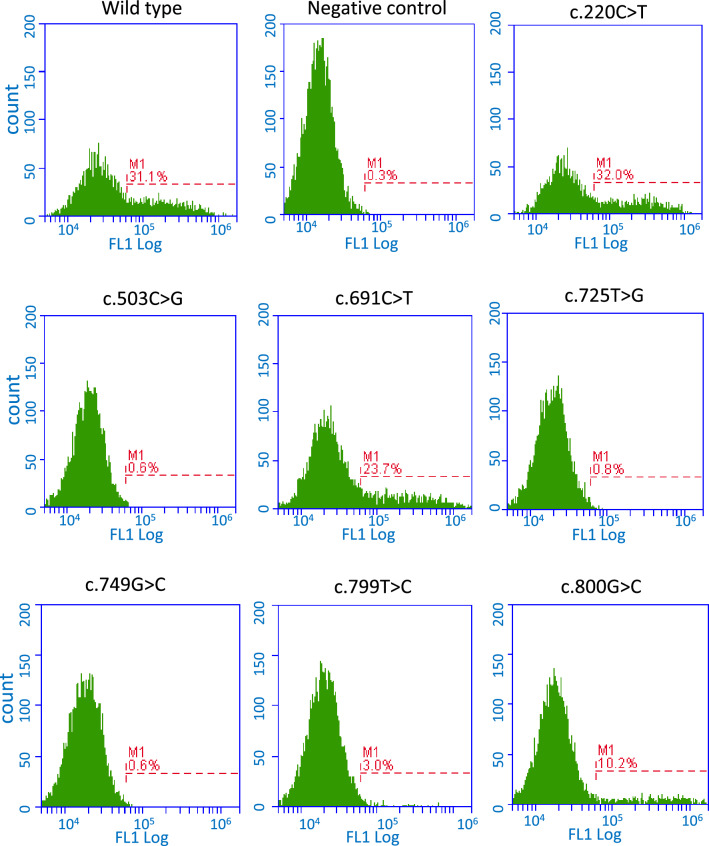
Expression of H antigens in the COS-7 cells transfected with various *FUT1* constructs. The *FUT1* allele containing uncharacterized SNP(s), wild-type allele (positive control), or FUT1*01N.09 (with c.725T>G) subcloned into pcDNA3.1(+) plasmids was transfected into COS-7 cells. Negative control is COS-7 cells transfected with pcDNA3.1(+) plasmid without *FUT1* allele. After 2 days of culture, the cells were incubated with 1E3 mouse monoclonal antibody to H type 1–4, followed by incubation with FITC-conjugated goat anti-mouse IgM secondary antibody, and expression of H antigen on the cell surface was monitored by flow cytometry. Nine representative flow cytometry results including positive and negative controls are shown.

For determination of whether each uncharacterized *FUT1* allele encodes a functional H enzyme or not, the α(1,2)fucosyltransferase activity in transfectants of each of *FUT1* expression vector was determined in previous studies^13,20^. In this study, we tried flow cytometry for measurement of H antigens expressed on the surface of culture cells using anti-H monoclonal antibody (1E3)^24^ because the phenotype of erythrocytes could not be demonstrated and antibody tests of serum could not be performed. The predicted amino acid change for each allele and the expression levels of H antigens on the cell surface are shown in Table 1. Nine representative flow cytometry results including positive and negative controls are shown in Fig. 2.

In this study, substitutions with significantly (p < 0.05) lower activity compared to the positive control (H antigen expression of wild-type *FUT1* allele, FUT1*01, 28.7 ± 3.2%) were defined as activity-affecting substitutions. Among them, alleles with substitutions whose activity was less than 10% of the positive control (H antigen expression of wildtype *FUT1* allele, FUT1*01) were considered weakly functional alleles. In addition, H-enzyme inactivating substitutions were defined as those with no difference in activity (p > 0.05) compared to the negative control (the expression level of pcDNA3.1(+) without insert, 0.7 ± 0.2%). The percentage of H antigen–positive cells transfected with pcDNA3.1 ligated with the *FUT1* of c.20G>C (p.R7P), c.101A>G (p.H34R), c.35C>T (p.A12V) and c.181G>A (p.A61T), c.35C>T (p.A12V) and c.220C>T (p.P74S), c.229C>G (p.L77V), c.283C>G (p.Q95E), c.468C>G (p.D156E), c.35C>T (p.A12V) and c.530T>G (p.L177R), c.565G>C (p.D189H), c.607C>T (p.R203C), c.625G>A (p.D209N), c.35C>T (p.A12V) and c.649G>A (p.V217I), c.796G>C (p.E266Q), c.1064A>G (p.D355G), and c.1096T>C (plus10 amino acids) were not significantly different (p > 0.05) with those ligated with the positive control of the wild-type allele, FUT1*01 (28.7 ± 3.2%). On the other hand, the percentage of H antigen–positive cells transfected with pcDNA3.1 ligated with *FUT1* of c.691C>T (p.R231C), c.1013T>A (p.I338N), and c.1022C>T (p.P341L) were two-third of the positive control and those of c.800G>C (p.W267S, 10.0 ± 1.5%, Table 1) were less than half of the positive control but much higher than that of c.799T>C (p.W267R, 2.4 ± 0.9%). In the same experimental condition, expression of the H antigen on cells transfected with pcDNA3.1-*FUT1* of c.503C>G (p.P168R, 0.4 ± 0.2%) or c.749G>C (p.R250P, 0.5 ± 0.2%) was almost undetectable as was that of c.725T>G (p.L242R, 0.4 ± 0.3%) and pcDNA3.1 without *FUT1* (0.7 ± 0.2%).

### In silico analysis to estimate the significance of uncharacterized nonsynonymous SNPs

We also predicted the possible impacts of 22 amino acid substitutions on the structure and function of the encoded H enzyme using four software programs, while c.1096T>C was excluded from the analysis because it occurred on T of termination codon TGA and 10 amino acids were added to the C-terminus (Table 1).

The results of predictions were not always consistent with those of expression experiments. For example, it has already been reported that p.L242R (c.725T>G), the substitution responsible for the classical Indian Bombay phenotype, is an H-deficient allele but it was classified as medium by Mutation Assessor. Of 22 amino acids substitutions, the predicted effects were matched for all software and experiments for p.A61T (c.181G>A), p.D156E (c.468C>G), p.D209N (c.625G>A) as polymorphic, neutral, benign, and tolerated substitutions and p.P168R (c.503C>G), p.L242R (c.725T>G), p.R250P (c.749G>C), p.W267S (c.799T>C) and p.I338N (c.1013T>A) as disease-causing, medium or low, damaging, and affected substitutions. On the other hand, they were mismatched for the other 14 substitutions (Table 1). Estimated concordance rates between in vitro expression studies and in silico function predictions were 81.8%, 50.0%, 68.2%, and 59.1% for MutationTaster, MutationAssessor, PolyPhen-2, and SIFT, respectively. Like the *FUT2*-encodedenzyme (Se enzyme) analyses^19^, the software generally tended to overestimate the impacts of the nonsynonymous SNPs we tested here.

### FUT2 alleles link to nonfunctional FUT1 alleles

Finally, we identified two newly characterized completely nonfunctional alleles (with c.503C>G and c.749G>C) and one weakly functional allele (with c.799T>C) in this study. According to the 1000 Genomes Project Database, the Database of Genomic Variants (http://dgv.tcag.ca/dgv/app/variant?id=esv3644597&ref=GRCh38/hg38)^31^, one heterozygote of *FUT1* with c.503C>G (HG02789, Punjabi in Lahore, Pakistan) was a compound heterozygote of FUT2*01N.02 (nonfunctional *FUT2* alleles with c.428G>A nonsense substitution) and FUT2*0N.01 (accession number: v3644597, approximately 10-kb deletion including the entire *FUT2* coding region) and one heterozygote of *FUT1* with c.749G>C (NA21128, Gujarati Indians in Houston, Texas) was a homozygote of FUT2*01N.02 (Table 2). Therefore, *FUT1* with c.503C>G was estimated to link to FUT2*01N.02 or FUT2*0N.01 and *FUT1* with c.749G>C to FUT2*01N.02. Accordingly, homozygotes of these alleles are expected to be the Bombay phenotype because both nonfunctional *FUT1* alleles were linked to nonfunctional *FUT2* allele. On the other hand, one heterozygote of *FUT1* with c.799T>C (NA19095, Yoruba in Ibadan, Nigeria) was a functional *FUT2* homozygote. Therefore, homozygotes for this allele are considered to be of the secretor phenotype. Unfortunately, we cannot be certain because the phenotype has not been confirmed, but homozygotes for this weak-functional allele are likely to be the para-Bombay phenotype, regardless of the secretor phenotype (Table 2).

**Table 2:** Linkage of nonfunctional or weakly functional alleles of *FUT1* to alleles of *FUT2.*

Subject	Attribution	*FUT1*	*FUT2*	Deduced haplotype	Estimated phenotype
HG03919	Bangladesh	Functional *FUT1*/FUT1*01N.09	FUT2*0N.01/FUT2*01N.15	FUT1*01N.09–FUT2*0N.01	Bombay
HG04189	Bangladesh	Functional *FUT1*/FUT1*01N.09	FUT2*0N.01/FUT2*01N.02	FUT1*01N.09–FUT2*0N.01	Bombay
HG02733	Pakistan	Functional *FUT1*/FUT1*01N.09	Functional FUT2/FUT2*01N.02	FUT1*01N.09–Functional *FUT2* or FUT1*01N.09­ FUT2*01N.02	Para-Bombay or Bombay
Sinhalese	Sri Lanka	Functional *FUT1*/FUT1*01N.09	Functional FUT2/FUT2*0N.01	FUT1*01N.09–FUT2*0N.01	Bombay
Tamil	Sri Lanka	Functional *FUT1*/FUT1*01N.09	FUT2*0N.01/FUT2*0N.01	FUT1*01N.09–FUT2*0N.01	Bombay
Bangladeshi	Bangladesh	Functional *FUT1*/FUT1*01N.09	FUT2*0N.01/FUT2*01N.15	FUT1*01N.09–FUT2*0N.01	Bombay
HG02789	Pakistan	Functional *FUT1*/*FUT1* with c.503C>G	FUT2*0N.01/FUT2*01N.02	*FUT1* with c.503C>G–FUT2*0N.01 or *FUT1* with c.503C>G–FUT2*01N.02	Bombay
NA21128	Texas (Gujarati)	Functional *FUT1*/*FUT1* with c.749G>C	FUT2*01N.02/ FUT2*01N.02	*FUT1* with c.749G>C–FUT2*01N.02	Bombay
NA19095	Nigerian	Functional *FUT1*/*FUT1* with c.749T>C	Functional *FUT2/* Functional *FUT2*	*FUT1* with c.749T>C–Functional *FUT2*	Para-Bombay

FUT1*01N.09: nonfunctional *FUT1* alleles with c.725T>G missense substitution. FUT2*0N.01: approximately 10-kb deletion including the entire *FUT2* coding region. FUT2*01N.15: nonfunctional *FUT2* alleles with c.302C>T missense substitution. FUT2*01N.02: nonfunctional *FUT2* alleles with 428G>A nonsense substitution. Estimated phenotype: estimated phenotype of homozygotes for each deduced haplotype.

The FUT1*01N.09 is known to links to FUT2*0N.01 in the classical Indian Bombay phenotype^20,21^. In fact, two FUT1*01N.09 heterozygotes (HG03919 and HG04189, both Bengalis in Bangladesh) were heterozygous for FUT2*0N.01 and these two FUT1*01N.09 alleles are presumed to link to FUT2*0N.01 (Table 2). However, one FUT1*01N.09 heterozygote (HG02733, Punjabi in Lahore, Pakistan) was functional *FUT2*/FUT2*01N.02 for the *FUT2*. Thus, in this one subject, FUT1*01N.09 does not link to FUT2*0N.01, and if it linked to functional *FUT2*, the homozygote for this allele would be a para-Bombay phenotype (Table 2).

### Screening of c.503C>G, c.725T>G, and c.749G>C by HRM in South Asian populations

**Figure 3:**
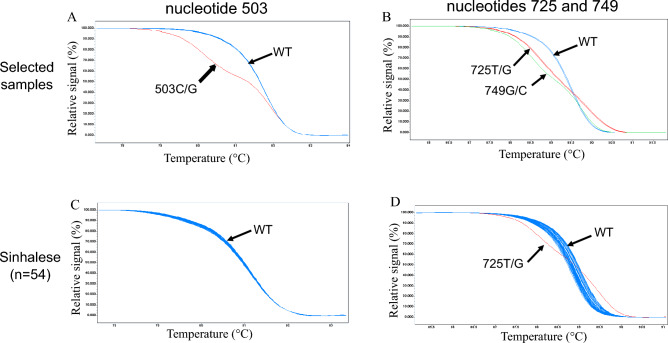
(**A**) Normalized and temperature-shifted melting curves for genotyping of c.503C>G. The six selected subjects having the wild type (WT) genotype c.503C/C, (blue) and one selected subject with the genotype c.503C/G (red) were completely separated. (**B**) Normalized melting curves for genotyping of c.725T>G and c.749G>C. Two selected subjects having WT genotypes (c.725T/T and c.749G/G, blue), two selected subjects (labelled 725T/G) with the genotype c.725T/G and c.749G/G (red), and one subject (labelled 749G/C) with the genotype c. 725T/T and c.749G/C (green) were completely separated. (**C**) Normalized and temperature-shifted melting curves for genotyping of c.503C>G obtained for randomly selected Sinhalese people (n = 54). There was no subject with c.503C>G. (**D**) Normalized melting curves for genotyping of c.725T>G and c.749G>C obtained for Sinhalese. The subjects having WT genotypes (blue), and a subject labelled 725T/G as above, (red) were completely separated. There was no subject with the genotype c.725G/G and c.749G/C in this population.

According to the Erythrogene database, three H-deficient alleles with c.503C>G, c.725T>G, and c.749G>C were present only in South Asian populations. Therefore, we attempted to screen these substitutions by HRM in South Asian populations. HRM clearly separated each heterozygote of c.503C>G (Fig. 3A), c.725T>G, and c.749G>C from the respective wild-type homozygote (Fig. 3B). Temperature shifting was then performed using a temperature shift threshold of default setting (5%) for detection for 503C>G. On the other hand, temperature shifting was not performed for c.725T>G and c.749G>C to clearly distinguish c.725T>G from c.749G>C heterozygotes although the melting curve pattern of the wild type allele is broader than that of the temperature-shifted pattern (Fig. 3C,D).

We then screened 54 Sinhalese (Fig. 3C,D), 58 Tamils (not shown) in Sri Lanka, and 47 Bangladeshis (not shown) and found one heterozygous c.725T>G in each population. And one (Tamil) was homozygous for FUT2*0N.01 and two (Sinhalese and Bangladeshi) were heterozygous for FUT2*0N.01 (Table 2).

## Discussion

Haplotype determination of *FUT1* was relatively easy due to the small number of SNPs and the low coexistence of multiple SNPs on a single allele. Therefore, only four subjects requiring cloning of the *FUT1* coding region into a plasmid for haplotype identification. Sequencing the clones revealed that the haplotypes of only one of four alleles were different from that registered in the Erythrogene database. This result is different from *FUT2* in that we recently examined the genomic DNA of 18 unidentified alleles of *FUT2* in Erythrogene database and found that the combination of SNPs for some alleles differed from the database due to multiple SNPs on a single allele^19^.

In this study, we also performed transient expression studies of 22 uncharacterized *FUT1* alleles available from the Erythrogene database and found two nonfunctional alleles, one weakly functional allele, and four alleles partially reduced encoded H enzyme activity. Fifteen alleles appeared to encode H enzymes equivalent to the wild-type. The H-deficient phenotype is known to be very rare compared to the nonsecretor phenotype, which is present in about 25% of many populations^1,3^. Accordingly, the frequency of *FUT1* substitutions is low (26 of 29 alleles were 0.1% or less in the1000 Genomes Project Database). In addition to c.725T>G, a causal substitution of the classical Indian Bombay phenotype, they were the only two substitutions here that completely inactivated enzyme activity. It is interesting to note that all of these were found in South Asian populations, but the reason for this is not clear at present. Classical Indian Bombay subjects with FUT1*01N.09 and FUT2*0N.01 have been reported not only in Indians, but also Bangladeshis, Pakistanis, Sri Lankans, and even in West Asian Iranians^20,21,32,33^. Thus, the causal haplotype (FUT1*01N.09–FUT2*0N.01) of the classical Indian Bombay phenotype is presumed to be present with some frequency, albeit low, and to be widespread in a broad band of South Asian populations and certain West Asian populations, while the other two nonfunctional *FUT1* alleles (with c.503C>G and c.749G>C) may be restricted to relatively specific populations in South Asia. To investigate distribution of these substitutions and estimation of prevalence of Bombay or para-Bombay phenotypes, a large-scale analysis of the South Asian population is needed, and the HRM analysis used in this study is expected to be a good tool for this purpose.

The allele with c.649G>A resulting in p.V217I appears to be functional in the present transient expression studies although all in silico analyses suggest that this substitution has significant impact of encoded protein. On the other hand, the allele with c.649G>T resulting in p.V217F is listed as a weakly functional allele with the name of FUT1*01W.24 by the ISBT 018 H (FUT1FUT2) blood group alleles v6.1 31-MAR-2023. In addition, the *FUT1* with c.799T>C that produces p.W267R significantly reduced H enzyme activity, and the *FUT1* with c.800G>C that produces p.W267S also reduced enzyme activity by more than half compared to the wild-type enzyme. Thus, 217 V and 267W appeared to be important for H enzyme activity.

As mentioned above, in the classical Indian Bombay phenotype, FUT1*01N.09 links to FUT2*0N.01 in the literature^12,21^, and in at least two 1000 Genomes Project subjects and three Tamil, Sinhalese, and Bangladeshi subjects with FUT1*01N.09 also appear to be linked to FUT2*0N.01. However, we encountered here one FUT1*01N.09 not linked to FUT2*0N.01 because the genotype of *FUT2* of this subject was functional *FUT2*/FUT2*01N.02. It is difficult to determine the exact haplotypes of *FUT1* and *FUT2* in this subject because *FUT1* and *FUT2* are 35 kb apart on chromosome 19q13.3^6^. Based on gene frequencies, it is likely that the c.725T>G substitution of *FUT1* occurred on the chromosome with FUT2*0N.01 in South Asia. Therefore, although we cannot be certain, it is likely that FUT1*01N.09, which does not link to the FUT2*0N.01, arose by homologous recombination between chromosomes during meiosis. In any case, since only one allele has been analyzed so far, analysis of a large number of samples and family analyses will be necessary to estimate the mechanism of generation of this allele.

## Conclusion

We identified two nonfunctional *FUT1* alleles (with c.503C>G and c.749G>C) and one weak allele (with c.799T>C) in samples in the 1000 Genomes Project Database. To estimate the impact of each SNP, transient expression studies are desirable for analysis of *FUT1* as well as *FUT2*.

## Data Availability

The datasets used and/or analysed during the current study available from the corresponding author on reasonable request.

## References

[1] Daniels, G. in *Human Blood Groups* (ed Geoff Daniels) 11–95 (Wiley-Blackwell, 2013).

[2] Le Pendu J, Lemieux RU, Lambert F, Dalix AM, Oriol R (1982). Distribution of H type 1 and H type 2 antigenic determinants in human sera and saliva. Am. J. Hum. Genet..

[3] Oriol R, Candelier JJ, Mollicone R (2000). Molecular genetics of H. Vox Sang..

[4] Larsen RD, Ernst LK, Nair RP, Lowe JB (1990). Molecular cloning, sequence, and expression of a human GDP-L-fucose:beta-D-galactoside 2-alpha-L-fucosyltransferase cDNA that can form the H blood group antigen. Proc. Natl. Acad. Sci. USA.

[5] Kelly, R. J., Rouquier, S., Giorgi, D., Lennon, G. G. & Lowe, J. B. Sequence and expression of a candidate for the human Secretor blood group alpha(1,2)fucosyltransferase gene (FUT2). Homozygosity for an enzyme-inactivating nonsense mutation commonly correlates with the non-secretor phenotype. *J. Biol. Chem.* **270**, 4640–4649. 10.1074/jbc.270.9.4640 (1995). 10.1074/jbc.270.9.4640 7876235

[6] Rouquier, S., *et al.* Molecular cloning of a human genomic region containing the H blood group alpha(1,2)fucosyltransferase gene and two H locus-related DNA restriction fragments. Isolation of a candidate for the human Secretor blood group locus. *J. Biol. Chem.* **270**, 4632–4639. 10.1074/jbc.270.9.4632 (1995). 10.1074/jbc.270.9.4632 7876234

[7] Yu LC, Yang YH, Broadberry RE, Chen YH, Lin M (1997). Heterogeneity of the human H blood group alpha(1,2)fucosyltransferase gene among para-Bombay individuals. Vox Sang..

[8] Storry JR (2006). Identification of six new alleles at the FUT1 and FUT2 loci in ethnically diverse individuals with Bombay and Para-Bombay phenotypes. Transfusion.

[9] Scharberg EA, Olsen C, Bugert P (2016). The H blood group system. Immunohematology.

[10] Bhende YM (1952). A "new" blood group character related to the ABO system. Lancet.

[11] Davey RJ, Tourault MA, Holland PV (1978). The clinical significance of anti-H in an individual with the Oh (Bombay) phenotype. Transfusion.

[12] Koda, Y., Soejima, M. & Kimura, H. Structure and expression of H-type GDP-L-fucose:beta-D-galactoside 2-alpha-l-fucosyltransferase gene (FUT1). Two transcription start sites and alternative splicing generate several forms of FUT1 mRNA. *J. Biol. Chem.* **272**, 7501–7505. 10.1074/jbc.272.11.7501 (1997). 10.1074/jbc.272.11.7501 9054453

[13] Kelly RJ (1994). Molecular basis for H blood group deficiency in Bombay (Oh) and para-Bombay individuals. Proc. Natl. Acad. Sci. USA.

[14] Scharberg EA, Olsen C, Bugert P (2019). An update on the H blood group system. Immunohematology.

[15] Lei, H. *et al.* A para-Bombay blood group case associated with a novel FUT1 mutation c.361G>A. *Transfus. Med. Hemother.* **48**, 254–258. 10.1159/000513318 (2021). 10.1159/000513318 PMC8406362 34539321

[16] Bureau V (2001). Comparison of the three rat GDP-L-fucose:beta-D-galactoside 2-alpha-L-fucosyltransferases FTA FTB and FTC. Eur. J. Biochem..

[17] Moller M, Joud M, Storry JR, Olsson ML (2016). Erythrogene: a database for in-depth analysis of the extensive variation in 36 blood group systems in the 1000 Genomes Project. Blood Adv..

[18] Sudmant PH (2015). An integrated map of structural variation in 2,504 human genomes. Nature.

[19] Soejima, M. & Koda, Y. Survey and characterization of nonfunctional alleles of FUT2 in a database. *Sci. Rep.* **11**. 10.1038/s41598-021-82895-w (2021). 10.1038/s41598-021-82895-w PMC7862633 33542434

[20] Koda Y, Soejima M, Johnson PH, Smart E, Kimura H (1997). Missense mutation of FUT1 and deletion of FUT2 are responsible for Indian Bombay phenotype of ABO blood group system. Biochem. Biophys. Res. Commun..

[21] Fernandez-Mateos P (1998). Point mutations and deletion responsible for the Bombay H null and the Reunion H weak blood groups. Vox Sang..

[22] Pang H (2000). Two distinct Alu-mediated deletions of the human ABO-secretor (FUT2) locus in Samoan and Bangladeshi populations. Hum. Mutat..

[23] Soejima M, Koda Y (2005). Denaturing high-performance liquid chromatography-based genotyping and genetic variation of FUT2 in Sri Lanka. Transfusion.

[24] Nakajima T, Yazawa S, Miyazaki S, Furukawa K (1993). Immunochemical characterization of anti-H monoclonal antibodies obtained from a mouse immunized with human saliva. J. Immunol. Methods.

[25] Schwarz JM, Rodelsperger C, Schuelke M, Seelow D (2010). MutationTaster evaluates disease-causing potential of sequence alterations. Nat. Methods.

[26] Reva B, Antipin Y, Sander C (2011). Predicting the functional impact of protein mutations: Application to cancer genomics. Nucleic Acids Res..

[27] Sunyaev S (2001). Prediction of deleterious human alleles. Hum. Mol. Genet..

[28] Sim NL (2012). SIFT web server: Predicting effects of amino acid substitutions on proteins. Nucleic Acids Res..

[29] Erali M, Voelkerding KV, Wittwer CT (2008). High resolution melting applications for clinical laboratory medicine. Exp. Mol. Pathol..

[30] Untergasser A (2012). Primer3–new capabilities and interfaces. Nucleic Acids Res..

[31] MacDonald JR, Ziman R, Yuen RK, Feuk L, Scherer SW (2014). The Database of Genomic Variants: A curated collection of structural variation in the human genome. Nucleic Acids Res..

[32] Ringressi A, Cunsolo V, Malentacchi F, Pozzessere S (2018). Erythrocyte phenotype in a pregnant woman of Sri Lanka: Description of the case and complications related to communication problems. Ann Ist Super Sanita.

[33] Shahriyari F, Oodi A, Kenari FN, Shahabi M (2023). Identification of two novel FUT1 mutations in people with Bombay phenotype from Iran. Transfus. Apher. Sci..

